# *p53* expression status is associated with cancer-specific survival in stage III and high-risk stage II colorectal cancer patients treated with oxaliplatin-based adjuvant chemotherapy

**DOI:** 10.1038/s41416-019-0429-2

**Published:** 2019-03-21

**Authors:** Hyeon Jeong Oh, Jeong Mo Bae, Xianyu Wen, Seorin Jung, Younghoon Kim, Kyung Ju Kim, Nam-Yun Cho, Jung Ho Kim, Sae-Won Han, Tae-You Kim, Gyeong Hoon Kang

**Affiliations:** 1 Department of Pathology, Seoul National University College of Medicine, Seoul, South Korea; 2 Laboratory of Epigenetics, Cancer Research Institute, Seoul National University College of Medicine, Seoul, South Korea; 3 Department of Internal Medicine, Seoul National University College of Medicine, Seoul, South Korea

**Keywords:** Predictive markers, Colorectal cancer, Predictive markers, Colorectal cancer, Predictive markers

## Abstract

**Background:**

We attempted to elucidate whether p53 expression or *TP53* mutation status was associated with cancer-specific survival in adjuvant FOLFOX-treated patients with stage III or high-risk stage II colorectal cancer (CRC).

**Methods:**

We analysed CRCs (*N* = 621) for the presence of *TP53* alterations and for p53 expression, using targeted resequencing and immunohistochemistry. CRCs were grouped into four subsets according to the p53 expression status, which included p53-no, mild, moderate and strong expression.

**Results:**

The distributions of CRCs were 19.85, 11.05, 17.7% and 51.5% in the p53-no, mild, moderate and strong expression groups, respectively. Cases in the p53-mild to moderate expression group were associated with a more frequent proximal location, undifferentiated histology, lower N category, extraglandular mucin production, microsatellite instability, CIMP-P1, CK7 expression and decreased CDX2 expression compared with those of cases of the p53-no expression and p53-strong expression groups. According to survival analysis, the p53-mild expression group showed a poor 5-year relapse-free survival (hazard ratio (HR): 2.71, 95% confidence interval (CI) = 1.60–4.60, *P* < 0.001) and poor 5-year cancer-specific survival (HR: 2.90, 95% CI = 1.28–6.57, *P* = 0.011).

**Conclusions:**

p53-mild expression status was found to be an independent prognostic marker in adjuvant FOLFOX-treated patients with stage III and high-risk stage II CRC.

## Background

For the last three decades, 5-fluorouracil (5-FU) has been the primary agent used in the treatment of patients with colorectal cancer (CRC) in both the adjuvant and palliative setting.^1,2^ An additional chemotherapeutic agent, oxaliplatin, has been approved for the treatment of CRC, and FOLFOX, a combination of 5-FU and oxaliplatin, led to increased responses and has become the standard of care in the adjuvant setting for patients with stage III or high-risk stage II CRC.^3,4^ However, a significant proportion of stage III patients receive adjuvant chemotherapy without benefit.^5^ The identification of biomarkers that predict tumour response to 5-FU-based chemotherapy is important for the personalised treatment of CRC, which will enhance tumour response and survival rates. However, despite the significant expenditure of efforts over two decades in search of biomarkers that predict tumour response to 5-FU-based chemotherapy, no clinically applicable biomarkers have been developed to predict chemotherapy benefit.^6,7^

p53 protein expression is a biomarker that is most frequently investigated for its predictive value in CRCs. p53 is induced by cellular stress, including DNA damage, shortened telomeres, hypoxia, aberrant growth signals and chemotherapy.^8^ p53 activated by DNA-damaging agents leads to cell cycle arrest in G1 phase and induction of DNA repair by transcriptional upregulation of the *CDKN1A* (*p21*) and *GADD45* genes. Studies in CRC cell lines and those derived from other carcinomas have suggested that the reaction to chemotherapy depends on whether the *TP53* gene is wild type or a mutant. The presence of wild-type *TP53* in cell lines was associated with in vitro growth inhibition in response to many chemotherapeutic agents, including DNA/RNA antimetabolites, alkylating agents and topoisomerase I and II inhibitors.^9–11^ Although *TP53* is a tumour-suppressor gene, *TP53* mutations can be either ‘gain of function' (GOF) mutations or ‘loss of function' (LOF) mutations. As efficient GOF action by mutant *TP53* requires the accumulation of mutant p53 in the affected cell, the determination of p53 overexpression using immunohistochemistry could suggest the presence of GOF mutations in *TP53*. In contrast, no detectable p53 in tumour cells indicates the presence of an LOF mutation in *TP53*. Thus, the different responses of CRCs to adjuvant chemotherapy depend on the expression status of p53. Many studies have attempted to correlate p53 expression status with prognosis or therapeutic response in CRC but have yielded inconsistent results regarding those relationships.^12–19^ However, the cutoff values that define p53 overexpression according to immunohistochemistry are variable among these studies. Furthermore, only a few studies have investigated the loss of p53 expression in tumour cells (no expression in tumour cells) as a predictive or prognostic biomarker.^20^

The purpose of the present study was to test the hypothesis that responses to adjuvant chemotherapy (FOLFOX) might differ in CRCs depending on the expression status of p53. We analysed 621 cases of stage III and high-risk stage II CRCs for p53 expression using immunohistochemistry and correlated the p53 expression status with the 5-year relapse-free survival and cancer-specific survival. Moreover, using the targeted exome-sequencing data of 469 patients, we compared the p53 expression status with the status of the *TP53*, *KRAS* and *BRAF* genes.^21^

## Methods

### Study population

In all, 655 patients with stage III or high-risk stage II CRC who received adjuvant fluoropyrimidine plus oxaliplatin after curative resection (R0) at Seoul National University Hospital between April 2005 and December 2012 were initially selected for this study. A total of 268 patients received FOLFOX-4, 276 patients received modified FOLFOX-6, and 111 patients received capecitabine plus oxaliplatin (XELOX). Adjuvant FOLFOX and XELOX were planned for a total of 12 and 8 cycles, respectively. The main inclusion criteria for the retrospective selection of patients were as follows: over 18 years of age, adenocarcinoma histology, stage III or high-risk stage II CRC, complete resection of the tumour with negative margins and the completion of at least six cycles of adjuvant FOLFOX chemotherapy or four cycles of adjuvant XELOX therapy. The criteria for high-risk stage II CRC were as follows: T4 lesion, obstruction or perforation, poorly differentiated histology and lymphovascular invasion or perineural invasion. We excluded the patients who received pre-operative radiotherapy and/or chemotherapy, and patients with a history of other malignancies within 5 years. Among them, 621 cases with p53 immunohistochemistry data were finally selected for this study. Demographic data and clinicopathological information were retrieved from electronic medical records. One pathologist (JMB) reviewed the haematoxylin and eosin-stained tissue slides to determine the tumour differentiation and extracellular mucin production. Disease stage was classified according to the seventh edition of the guidelines of the American Joint Committee on Cancer (AJCC).

### DNA extraction, MSI and DNA methylation analyses

After microscopic examination of the haematoxylin and eosin-stained slides from formalin-fixed paraffin-embedded (FFPE) tissues, areas that represented the primary histology and the highest tumour to non-tumour cell ratio were selected and scraped from the slides with knife blades. The scraped tissues were collected into microtubes containing lysis buffer (50 mm Tris, 1 mm ethylenediaminetetraacetic acid, pH 8.0 and 1 % Tween-20) and proteinase K (3 mg/mL); the microtubes were then incubated at 55 ℃ for up to 2 days. After centrifugation, the supernatants were transferred into newly labelled microtubes. The samples were then placed into a 95 °C heating block for 30 min to inactivate the proteinase K and to increase the accuracy of the DNA methylation analysis of formalin-fixed tissue samples. Microsatellite instability (MSI) was assessed at the following loci: BAT25, BAT26, D2S123, D5S346 and D17S250. Samples were classified as microsatellite instability-high (MSI-H) when at least 40% of loci showed MSI. Otherwise, samples were classified as MSI-low/microsatellite stable (MSI-L/MSS). The lysed tissue solution was subjected to a DNA bisulphite modification, which was performed as previously described.^22^ Using a real-time PCR-based methylation assay (MethyLight), we quantified DNA methylation in eight CIMP panel markers which included *CACNA1G*, *CDKN2A*, *CRABP1*, *IGF*2, *MLH1*, *NEUROG1*, *RUNX3* and *SOCS1*. The primer sequences and PCR conditions have also been previously described.^23,24^ Tumours were regarded as CIMP-negative (CIMP-N, 0–4 methylated genes), CIMP-positive 1 (5–6 methylated genes) and CIMP-positive 2 (7–8 methylated genes), as previously described.^25^

### Immunohistochemistry

A tissue microarray (TMA) was constructed using FFPE tissues from 621 CRCs. Two different tumour areas were sampled as tissue cores (2 mm in diameter) and were transferred to the TMA blocks. Immunohistochemical analyses were performed with commercially available antibodies against p53 (clone DO-7; 1:200), cytokeratin 7 (CK7, clone OV-TL 12/30; DAKO), cytokeratin 20 (CK20; clone K20.8, DAKO), CDX2 (clone EPR2764Y ready-to-use, Cell Marque), p21 (clone DCS-60.2, Cell Marque) and cyclin D1 (clone SP4, Thermo Fisher). All the immunohistochemistry slides were scanned using an Aperio ScanScope CS (Aperio Technologies, Inc., Vista, CA, USA). For the quantitative analysis of p53 immunohistochemistry, the proportion of tumour cells with moderate-to-strong nuclear staining (intensity scores of 2 + to 3 + ) for p53 was measured by the Image Scope computerised image analysis system (Aperio Technologies) using the Nuclear v9 algorithm (Supplementary figure 1). Then, expression in the tumours was defined as p53-no, p53-weak, p53-moderate, or p53-strong when < 1%, 1–10%, 10–50% or ≥ 50% of tumour cells showed p53 immunoreactivity, respectively. The immunohistochemistry results for CK7, CK20 and CDX2 were obtained from a previous study.^25^ The quantitative analysis of nuclear p21 and cyclin D1 expression were evaluated using QuPath (https://qupath.github.io).^26^ The nuclear intensities of tumour cells were divided into four groups of negative, weak, moderate and strong, based on the average nuclear diaminobenzidine optical density. Cell number of each group was measured through *Cell detection* command of QuPath. Expression rates were calculated as the percentage of the number of positive (weak, moderate and strong) cells to the total number of tumour cells.

### Next-generation sequencing

Of the 621 cases, 469 cases were included in our previous study in which targeted exome sequencing of 40 genes was performed using a HiSeq2500 (Illumina, USA) by Celemics, Inc. (Seoul, South Korea).^21^ The sequencing results of the *TP53*, *KRAS* and *BRAF* genes were obtained from that study. In brief, areas of the tumour with the highest tumour purity were dissected from unstained serial sections of FFPE tissues, methacarn-fixed paraffin-embedded tissues, or fresh frozen tissues. Sheared genomic DNA ( > 200 ng) was prepared according to the routine library preparation process including end-repair, A-tailing and adapter ligation. The target enrichment process proceeded based on in-solution hybridisation with biotinylated probes. Sequencing data were mapped to the human GRCh37 using bwa mem version 0.7.5a. Aligned reads were processed with Picard MarkDuplicates and the Genome Analysis Toolkit after base recalibration. After a series of processes, aligned bases were collected using SAMtools. Somatic variant calling and annotation were performed using VarScan and ANNOVAR, respectively. For variants with COSMIC IDs, variants with altered reads > 10 and those with a variant allele frequency (VAF) > 5% were included. For variants without COSMIC IDs, variants with altered reads > 20 and those with a VAF > 10% were included.

### Statistical analysis

The clinical database was last updated in July 2016. The cancer-specific survival (CSS) time was calculated from the date of surgery to the date of death from CRC. The relapse-free survival (RFS) time was calculated from the first date of chemotherapy to the date of documented relapse. The data of patients who did not experience cancer-specific death or relapse were censored at the date of the last follow-up visit to obtain the CSS and RFS. Differences in distributions between the parameters examined were assessed using the Chi-square or Fisher’s exact test, as appropriate. CSS and RFS were calculated using the log-rank test with Kaplan–Meier curves. The Cox-proportional hazard model was used for the multivariate survival analysis with adjustments for variables that were significant (*P* < 0.05) or marginally significant (0.05 ≤ *P* < 0.10) prognostic factors according to the univariate analysis. All statistical tests were two-sided, and statistical significance was defined as *P* < 0.05.

## Results

### Clinicopathological characteristics

In all, 621 patients with stage III or high-risk stage II CRC were included. The male-to-female ratio was 1.49:1 (372 males and 249 females), and the median age was 60 years (range, 29–78 years). The tumour location was the caecum in 25 patients, ascending colon in 139, hepatic flexure in 3, transverse colon in 39, splenic flexure in 1, descending colon in 36, sigmoid colon in 307, rectosigmoid colon in 29 and the upper rectum in 42 patients. Ninety-eight patients (15.8%) were diagnosed with high-risk stage II disease, while 523 patients (84.2%) were diagnosed with stage III disease.

### Clinicopathologic characteristics of CRCs according to p53 expression status or TP53 genotype

**Fig. 1:**
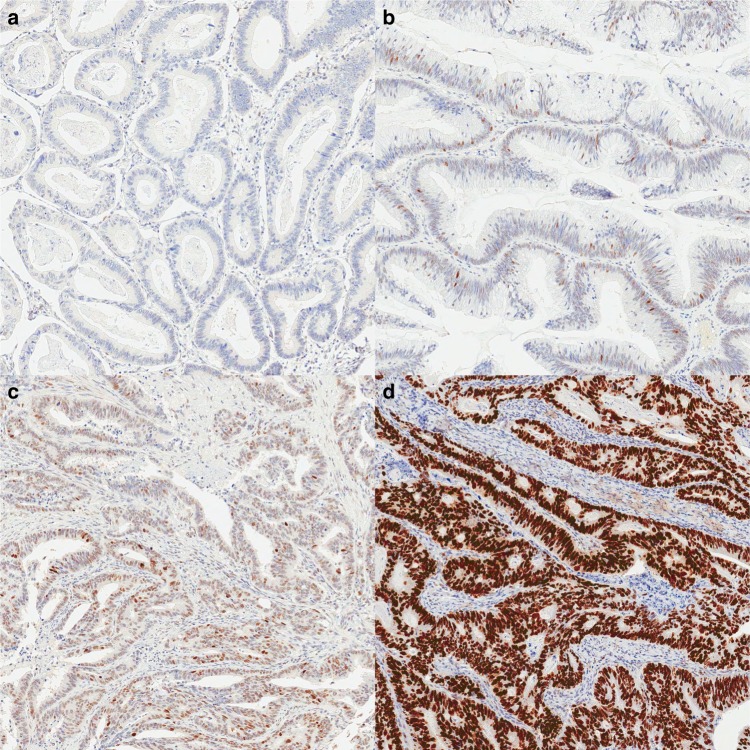
p53 immunohistochemistry, **a** p53-no expression, **b** p53-mild expression, **c** p53-moderate expression and **d** p53-strong expression

**Fig. 2:**
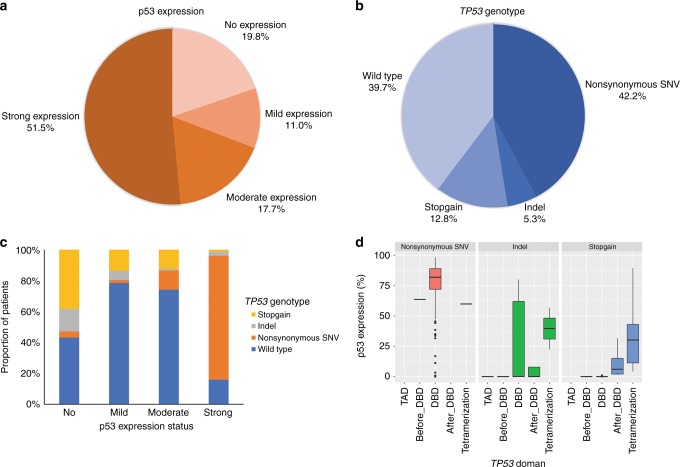
Correlation between p53 expression and TP53 genotype. **a** Proportion of four pTP53 expression subgroups, **b** proportion of each TP53 genotype, **c** proportion of TP53 genotypes in the four p53 expression subgroups and **d** pTP53 expression status according to the location of genetic alterations in the TP53 gene

The clinicopathologic characteristics of CRCs according to p53 expression status and *TP53* genotype are summarised in Tables 1 and 2. p53-no, p53-mild, p53-moderate and p53-strong expression was found in 123 (19.8%), 68 (11.0%), 110 (17.7%) and 320 (51.5%) patients, respectively (Figs. 1 and 2a). Among the four different p53 expression groups, the p53-mild and p53-moderate expression groups were associated with proximal location (*P* < 0.001), undifferentiated histology (*P* < 0.001), advanced N category (*P* = 0.007), higher stage (*P* = 0.001) and extraglandular mucin production (*P* < 0.001) compared with the p53-no and p53-strong expression groups. In terms of the molecular aspect, the p53-mild and p53-moderate expression groups were associated with MSI-H (*P* < 0.001), high frequency of CIMP-P1 or CIMP-P2 (*P* < 0.001), *MLH1* methylation (*P* < 0.001), aberrant CK7 expression (*P* = 0.013) and decreased CDX2 expression (*P* < 0.001).

**Table 1:** Clinicopathological features of colorectal cancers according to the p53 expression status

	p53-no expression (*N*=123, 19.8%)	p53-weak expression (*N*=68, 11.0%)	p53-moderate expression (*N*=110, 17.7%)	p53-strong expression (*N*=320, 51.5%)	*P*
Age, median (min–max)	61 (30–75)	59 (29–78)	62 (31–76)	60 (30–78)	0.956
Sex					0.304
Male	79 (64.2%)	43 (63.2%)	58 (52.7%)	192 (60.0%)	
Female	44 (35.8%)	25 (36.8%)	52 (47.3%)	128 (40.0%)	
Location					< 0.001
Proximal colon	33 (26.8%)	32 (47.1%)	56 (50.9%)	85 (26.6%)	
Distal colon, rectum	90 (73.2%)	36 (52.9%)	54 (49.1%)	235 (73.4%)	
Gross pattern					0.448
Fungating	73 (59.3%)	46 (67.6%)	72 (65.4%)	190 (59.4%)	
Ulcerative	50 (40.7%)	22 (22.4%)	38 (34.6%)	130 (40.6%)	
Differentiation					< 0.001
Differentiated	121 (98.4%)	54 (79.4%)	92 (83.6%)	307 (95.9%)	
Undifferentiated	2 (1.6%)	14 (20.6%)	18 (16.4%)	13 (4.1%)	
T category					0.818
T1–3	103 (83.7%)	56 (82.3%)	94 (85.5%)	276 (86.2%)	
T4	20 (16.3%)	12 (17.7%)	16 (14.5%)	44 (13.8%)	
N category					< 0.001
N0, N1	80 (65.0%)	56 (82.4%)	91 (82.7%)	233 (72.8%)	
N2	43 (35.0%)	12 (17.6%)	19 (17.3%)	87 (27.2%)	
Stage					0.001
II, high-risk	10 (8.1%)	15 (22.1%)	28 (25.5%)	45 (14.1%)	
III	113 (91.9%)	53 (77.9%)	82 (74.5%)	275 (85.9%)	
Lymphovascular invasion					0.168
Absent	72 (58.5%)	41 (60.3%)	60 (54.5%)	157 (49.1%)	
Present	51 (41.5%)	27 (39.7%)	50 (45.5%)	163 (50.9%)	
Perineural invasion					0.692
Absent	86 (69.9%)	51 (75.0%)	86 (78.2%)	231 (72.2%)	
Present	37 (30.1%)	17 (25.0%)	24 (21.8%)	89 (27.8%)	
Extraglandular mucin production				< 0.001	
Absent	119 (96.7%)	53 (77.9%)	82 (74.5%)	306 (95.6%)	
Present	4 (3.3%)	15 (22.1%)	28 (25.5%)	14 (4.4%)	
Microsatellite instability					< 0.001
MSS, MSI-L	118 (95.9%)	50 (74.6%)	93 (86.1%)	311 (97.8%)	
MSI-H	2 (1.7%)	17 (25.4%)	15 (13.9%)	7 (2.2%)	
CIMP					< 0.001
CIMP-N	118 (97.5%)	57 (85.1%)	94 (86.2%)	304 (95.6%)	
CIMP-P1	3 (2.5%)	7 (10.4%)	9 (8.3%)	12 (3.8%)	
CIMP-P2	0 (0.0%)	3 (4.5%)	6 (5.5%)	2 (0.6%)	
*MLH1* methylation					< 0.001
Unmethylated	120 (99.2%)	58 (86.6%)	100 (91.7%)	314 (98.7%)	
Methylated	1 (0.8%)	9 (13.4%)	9 (8.3%)	4 (1.3%)	
CK7 expression					0.013
Not expressed	119 (96.7%)	61 (89.7%)	97 (88.2%)	305 (95.3%)	
Expressed	4 (3.3%)	7 (10.3%)	13 (11.8%)	15 (4.7%)	
CK20 expression					0.065
Retained	112 (91.1%)	56 (82.3%)	91 (82.7%)	288 (90.0%)	
Decreased	11 (8.9%)	12 (17.7%)	19 (17.3%)	32 (10.0%)	
CDX2 expression					< 0.001
Retained	116 (94.3%)	53 (77.9%)	92 (83.6%)	296 (92.5%)	
Decreased	7 (5.7%)	15 (22.1%)	18 (16.4%)	24 (7.5%)

**Table 2:** Clinicopathological features of colorectal cancers according to the TP53 gene status (*N* = 469)

	*TP53* wild type (*N*=186, 39.7%)	*TP53* mutant type (*N*=283, 60.3%)	*P*
Age, median (min–max)	61 (29–76)	60 (30–78)	0.856
Sex			0.351
Male	105 (56.4%)	172 (60.8%)	
Female	81 (43.6%)	111 (39.2%)	
Location			0.021
Proximal colon	73 (39.3%)	82 (29.0%)	
Distal colon, rectum	113 (60.7%)	201 (71.0%)	
Gross type			0.002
Fungating	133 (71.5%)	163 (57.6%)	
Ulcerative	53 (28.5%)	120 (42.4%)	
Differentiation			0.017
Differentiated	165 (88.7%)	268 (94.7%)	
Undifferentiated	21 (11.3%)	15 (5.3%)	
T category			0.804
T1–3	158 (84.9%)	238 (84.1%)	
	28 (15.1%)	45 (15.9%)	
N category			0.032
N0, N1	145 (78.0%)	195 (68.9%)	
N2	41 (22.0%)	88 (31.1%)	
Stage			0.143
II, high-risk	35 (18.8%)	39 (13.8%)	
III	151 (81.2%)	244 (86.2%)	
Lymphovascular invasion			0.321
Absent	104 (55.9%)	145 (51.2%)	
Present	82 (44.1%)	138 (48.8%)	
Perineural invasion			0.067
Absent	145 (78.0%)	199 (70.3%)	
Present	41 (22.0%)	84 (29.7%)	
Extraglandular mucin			< 0.001
Absent	149 (80.1%)	269 (95.0%)	
Present	37 (19.9%)	14 (5.0%)	
Microsatellite instability (*N* = 463)			< 0.001
MSS, MSI-L	154 (83.7%)	275 (98.6%)	
MSI-H	30 (16.3%)	4 (1.4%)	
CpG island methylator phenotype (*N* = 464)			0.006
CIMP-N	164 (89.1%)	268 (95.7%)	
CIMP-P1	11 (6.0%)	10 (3.6%)	
CIMP-P2	9 (4.9%)	2 (0.7%)	
*MLH1* methylation (*N* = 464)			0.008
Unmethylated	172 (93.5%)	275 (98.2%)	
Methylated	12 (6.5%)	5 (1.8%)	
CK7 expression			0.282
Not expressed	170 (91.4%)	266 (94.0%)	
Expressed	16 (8.6%)	17 (6.0%)	
CK20 expression			0.033
Retained	156 (83.9%)	256 (90.5%)	
Decreased	30 (16.1%)	27 (9.5%)	
CDX2 expression			0.188
Retained	161 (86.6%)	256 (90.5%)	
Decreased	25 (13.4%)	27 (9.5%)	
*KRAS* gene status			0.034
Wild type	98 (52.7%)	177 (62.5%)	
Mutant type	88 (47.3%)	106 (37.5%)	
*BRAF* V600E			0.100
Wild type	176 (94.6%)	276 (97.5%)	
Mutant type	10 (5.4%)	7 (2.5%)

Among the 469 CRC patients with targeted exome-sequencing data, 198 patients (42.2%) showed nonsynonymous SNVs, 25 patients (5.3%) showed indels, 60 patients (12.8%) showed stop–gain mutations and 186 patients (39.7%) had wild-type *TP53* (Fig. 2b). Mutant *TP53* was associated with proximal location (*P* = 0.021), gross ulcerative pattern (*P* = 0.002), differentiated histology (*P* = 0.017), advanced N category (*P* = 0.032), less extraglandular mucin production (*P* < 0.001), less frequent MSI-H (*P* < 0.001), *KRAS* mutations (*P* = 0.034), CIMP-P1 or CIMP-P2 (*P* = 0.006), *MLH1* methylation (*P* = 0.008) and decreased CK20 expression (*P* = 0.033) compared with wild-type *TP53*.

### Correlation of p53 expression status with the TP53 genotype

To explore the correlation of p53 expression with the *TP53* genetic status, we compared the p53 immunohistochemistry results with the targeted exome-sequencing data (Fig. 2c). In the p53-no expression group, stop–gain mutations were found in 39 patients (38.2%) and indels were found in 15 patients (14.7%). In the p53-strong expression group, nonsynonymous SNVs were found in 198 patients (80.2%). The p53-mild expression group showed fewer nonsynonymous SNVs compared with the p53-moderate expression group (2.0% vs. 12.4%).

As the *TP53* gene is composed of several functional domains, we compared p53 expression status with the nucleotide position of each type of mutation (Fig. 2d). For nonsynonymous SNVs, mutations in the DNA-binding domain (DBD) showed higher p53 expression compared with mutations in other domains. In terms of stop–gain mutations, tumours with mutations in the transactivation domain to the DBD showed no p53 expression; however, tumours with mutations behind the DBD showed mild to moderate p53 expression.

### Survival analysis

**Fig. 3:**
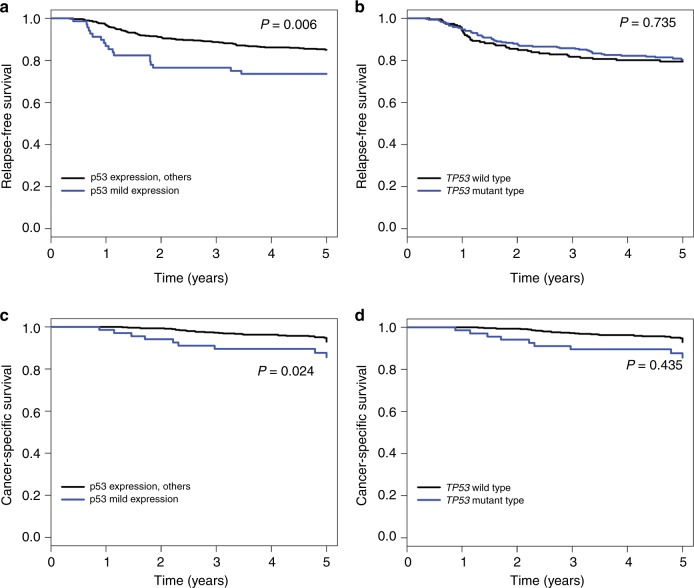
Kaplan–Meier survival curves. **a** Five-year relapse-free survival according to p53 expression, **b** 5-year RFS according to TP53 mutation status, **c** 5-year cancer-specific survival according to p53 expression and **d** 5-year CSS according to TP53 mutation status

To elucidate whether p53 expression status or *TP53* genotype is associated with clinical outcomes of CRC patients treated with FOLFOX or XELOX, we performed univariate and multivariate survival analyses. In the univariate survival analysis, the p53-mild expression group exhibited the worst 5-year RFS (*P* = 0.006) (Fig. 3a and Supplementary figure 2A) and 5-year CSS (*P* = 0.024) (Fig. 3c and Supplementary figure 2C) compared with the p53-no, p53-moderate and p53-strong expression groups. However, the 5-year RFS and the 5-year CSS were not significantly different according to the *TP53* genotype (Fig. 3b, d, Supplementary figure 2B and 2D). In the multivariate analysis, the p53-mild expression group exhibited a worse 5-year RFS (hazard ratio (HR) = 2.71, 95% confidence interval (CI) 1.21–3.36, *P* < 0.001) and 5-year CSS (HR = 2.90, 95% CI 1.28–6.57, *P* = 0.011) compared with CRCs in the p53-no, p53-moderate and p53-strong expression groups (Table 3 & Supplementary table 1).

**Table 3:** Cox-proportional hazard model for 5-year relapse-free survival

Variables	Univariate		Multivariate	
	HR (95% CI)	*P*	HR (95% CI)	*P*
Age ( ≥ 62/ < 62)	0.99 (0.67–1.47)	0.959		
Sex (male/female)	1.10 (0.73–1.65)	0.653		
Location (proximal colon/distal colon, rectum)	0.92 (0.61–1.41)	0.717		
Gross type (ulcerative/fungating)	1.73 (1.16–2.56)	0.007	–	0.434
Differentiation (undifferentiated/differentiated)	2.36 (1.34–4.16)	0.003	–	0.108
T category (T4/T1–3)	2.32 (1.48–3.63)	< 0.001	2.16 (1.38–3.40)	0.001
N category (N2/N0, N1)	2.62 (1.76–3.89)	< 0.001	2.53 (1.68–3.83)	< 0.001
Lymphovascular invasion (present/absent)	2.13 (1.42–3.21)	< 0.001	1.82 (1.19–2.78)	0.006
Perineural invasion (present/absent)	2.05 (1.38–3.06)	< 0.001	1.73 (1.15–2.60)	0.008
Extraglandular mucin (present/absent)	1.64 (0.93–2.88)	0.088		0.079
Microsatellite instability (MSI-H/MSS, MSI-L)	0.96 (0.42–2.19)	0.916		
CIMP (CIMP-P1/CIMP-N, P2)	1.61 (0.75–3.48)	0.223		
CK7 (expressed/not expressed)	2.32 (1.27–4.24)	0.006	1.93 (1.05–3.55)	0.035
CK20 (decreased/retained)	0.55 (2.26–1.19)	0.127		
CDX2 (decreased/retained)	1.54 (0.87–2.71)	0.136		
p53 expression (weak/no, moderate, strong)	2.02 (1.21–3.36)	0.007	2.71 (1.60–4.60)	< 0.001
*TP53* gene status (mutant type/wild type)	0.92 (0.61–1.40)	0.921		
*KRAS* gene status (mutant type/wild type)	1.26 (0.84–1.90)	0.267		
*BRAF* gene status (mutant type/wild type)	1.57 (0.64–3.87)	0.326		

Because p53-mild expression group was enriched in MSI-H CRCs compared with microsatellite stable (MSS) or microsatellite instability-low (MSI-L) CRCs (41.5% in MSI-H CRCs vs. 8.7% of MSS/MSI-L CRCs), we compared prognostic value of p53 expression in MSI-H CRCs and MSS/MSI-L CRCs (Supplementary figure 4). We found that p53-mild expression group showed poor 5-year RFS and 5-year CSS, however, the number of MSI-H CRCs is too small to get meaningful conclusion.

Even though there were no significant clinicopathologic difference between p53-mild expression group and p53-moderate expression group, only p53-mild expression group showed poor 5-year RFS and 5-year CSS. To elucidate differential prognostic effect between p53-mild expression group and p53-moderate expression group, we evaluated nuclear expression of cell cycle regulator p21 and cyclin D1 (Supplementary figure 5). P53-moderate expression group showed significantly higher p21 and cyclin D1 expression compared with other groups. Moreover, p53-moderate expression group showed the highest proportion of p21 and cyclin D1 co-expressed tumours than other groups.

## Discussion

Owing to the central role of p53 in genomic stability, *TP53* is one of the most-commonly mutated genes in human cancers; the prevalence of *TP53* mutations exceeds 40% in colorectal, head and neck, and oesophageal cancers (http://p53.iarc.fr/RefsHighlights.aspx). For specific types of cancer in which the frequency of *TP53* mutations is < 20%, p53 can be inactivated by protein interactions with Mdm2, Mdm4 or Twist.^27,28^ Clues that p53 is a tumour suppressor stem from earlier studies showing that the loss of p53 promotes cancer^29^ and that the p53 protein suppresses growth and oncogenic transformation in cell culture.^30^ The role of p53 as a tumour suppressor is further evidenced by a hereditary cancer-predisposition syndrome, Li-Fraumeni syndrome, in which germline *TP53* mutations are largely responsible for a cancer-prone phenotype; experimental animal models in which p53 loss confers a cancer-prone phenotype have also been developed.^31^ The functional inequivalence of *TP53* mutants is evident from the variable onset and pathological findings of tumours in patients with Li-Fraumeni syndrome or in genetically modified mice depending on the type of *TP53* mutation.^32,33^ The majority of *TP53* mutations occur in the DNA-binding domain, which abrogates the sequence-specific DNA-binding activity of p53. However, many mutant p53 forms gain new oncogenic properties such as the promotion of invasion and metastasis and the inhibition of cell death.^34^ The GOF effect of mutant p53 might be derived from the binding to p53 family proteins, such as p63 and p73, and their subsequent inactivation. By inhibiting p63, mutant p53 can regulate the expression of proteins related to pro-invasive transcription programmes, such as Dicer, Depdc1, Cyclin G2 and Sharp1.^35^ In addition, p53 GOF mutants can also bind to chromatin-regulatory genes such as methyltransferase, *MLL1*, *MLL2,* and the acetyl transferase, *MOZ*, and promote activating histone modifications.^36^

The correlation between mutant *TP53* and p53 expression has been investigated in many studies. In a study of ovarian carcinoma (*n* = 76) in which the extent of p53 immunoreactivity was classified into three categories ( < 5% (low), 5–69% (intermediate) and ≥ 70% positively stained nuclei (high expression)), p53-high and p53-low expressions were strongly associated with missense and non-missense mutations in *TP53*, respectively.^37^ Although cancers with wild-type *TP53* showed a wide range of positively stained nuclei, most cancers with wild-type *TP53* show intermediate p53 expression.^37^ In a study that analysed triple-negative breast cancers (*n* = 172) for genetic and protein alterations in *TP53* using next-generation sequencing and immunohistochemistry, respectively, p53 expression status ( ≤ and > 10% of tumour cells with nuclear staining) was closely associated with *TP53* mutational status. Although indel and missense mutations were relatively more frequent in tumours with ≤ and > 10% nuclear staining, respectively, the wild-type genotype comprised 50% and 64% of tumours with ≤ and > 10% nuclear staining, respectively.^38^ In the present study, 53% of CRCs in the p53-no expression group had stop–gain mutations or indels, whereas 80% of CRCs in the p53-strong expression group were characterised by missense mutations, which suggests that the p53-no expression and p53-strong expression statuses are likely to represent loss of function and gain of function, respectively. More than 70% of CRCs in the p53-mild or p53-moderate expression groups did not have *TP53* mutations, which suggests that CRCs with mild or moderate p53 expression are more likely to represent CRCs with wild-type *TP53*. Although subgroups classified according to the extent of p53 immunoreactivity are heterogenous in terms of *TP53* genotype, three-tiered or four-tiered classification appears superior to two-tiered classification in terms of the correlation between p53 expression status and *TP53* genotype.

Regarding the impact of *TP53* alterations on the survival of patients with CRC treated with 5-FU-based adjuvant chemotherapy, it is generally agreed that the outcome of patients is not influenced by either p53 expression or *TP53* mutation status.^15–17,39,40^ However, despite the finding that oxaliplatin-based adjuvant chemotherapy is the current standard treatment for stage III colon cancer, only a few studies have analysed the prognostic impact of *TP53* alterations on CRC patients treated with this regimen.^12^ In the study by Zaanan et al.^12^ p53 overexpression ( > 50% of tumour cells with nuclear staining) was found to be an independent factor that predicts whether a patient with stage III colon cancer will benefit from adjuvant FOLFOX vs. 5-FU and leucovorin (FL). This suggests that the lack of p53 overexpression ( < 50% of tumour cells with nuclear staining) may predict no benefit from adjuvant FOLFOX vs. FL. However, tumours with no p53 overexpression were heterogeneous and could be further divided into the p53-no, p53-mild and p53-moderate expression groups according to the classification in the present study. In this study, CRCs with no p53 showed CSS and RFS curves similar to the respective curves of CRCs with moderate p53 or strong p53 expression (Supplementary Figure 2), whereas mild p53 expression in CRCs was found to be an independent parameter associated with shorter CSS and RFS. As adjuvant FL-treated CRC patients were not included in the present study, the p53 expression status was not able to be assessed for its predictability of responses against FOLFOX vs. FL. Our findings are in agreement with those of Zaanan et al.^19^ and suggest that p53-mild status might predict no benefit from FOLFOX vs. FL. In a previous study by the Ogino group in which a survival analysis was conducted in patients with stage I, II, III, or IV CRC according to p53 expression status ( < vs. ≥ 50% of tumour cells with nuclear staining), p53-positivity ( ≥ 50% of tumour cells with nuclear staining) was associated with poor prognosis in the multivariate analysis. However, when we divided our FOLFOX cohort patients into two subgroups in accordance with the Ogino group’s classification of p53 expression status, we did not find a difference in survival between the two subgroups (Supplementary Figure 3).^19^

p21, downstream effector of p53, has a key role as a modulator of cell cycle arrest by inhibiting cyclin D1/cyclin-dependent kinase 4/6 (CDK4/6) complexes in G1/S transition.^41^ On the other hand, Ioachim reported the positive relationship between p21 and cyclin D1 expression in CRCs.^42^ Moreover, Alt et al.^43^ reported that p21 induces nuclear accumulation of cyclin D1 by preventing nuclear export. These results suggest that the expression of p21 and cyclin D1 is affected reciprocally. In CRCs, the associations of p21 expression and better prognosis were reported in several studies, which is consistent with tumour inhibitory function of p21.^44,45^ The prognostic value of cyclin D1 in CRCs is still controversial.^46^ However, prospective studies by Ogino et al. and Belt et al.^47,48^ showed that cyclin D1 overexpression is associated with better clinical outcome in CRCs. Higher expression of p21 and cyclin D1 in p53-moderate expression group might be one of the reason why p53-moderate expression group showed better 5-year RFS and 5-year CSS compared with p53-mild expression group in our present study.

In summary, we found that of the four expression statuses of p53, mild expression of p53 was closely associated with a poor 5-year RFS and a poor 5-year CSS in stage III or high-risk stage II CRC patients treated with adjuvant FOLFOX or XELOX. Our present study has limitation from the retrospective nature in a single institution. The results of present study should be validated in independent prospective study sets.

## Supplementary information

Supplementary figure 1

Supplementary figure 2

Supplementary figure 3

Supplementary figure 4

Supplementary figure 5

Supplementary table 1

Article File

## Data Availability

The data sets used and/or analysed during this study are available from the corresponding author on reasonable request, and most of the original data are included in this article.
