# Supplementary figures and images for: p53 expression status is associated with cancer-specific survival in stage III and high-risk stage II colorectal cancer patients treated with oxaliplatin-based adjuvant chemotherapy
Source: Br J Cancer. 2019 Mar 21;120(8):797–805. doi: 10.1038/s41416-019-0429-2 (PMC6474280; doi:10.1038/s41416-019-0429-2)

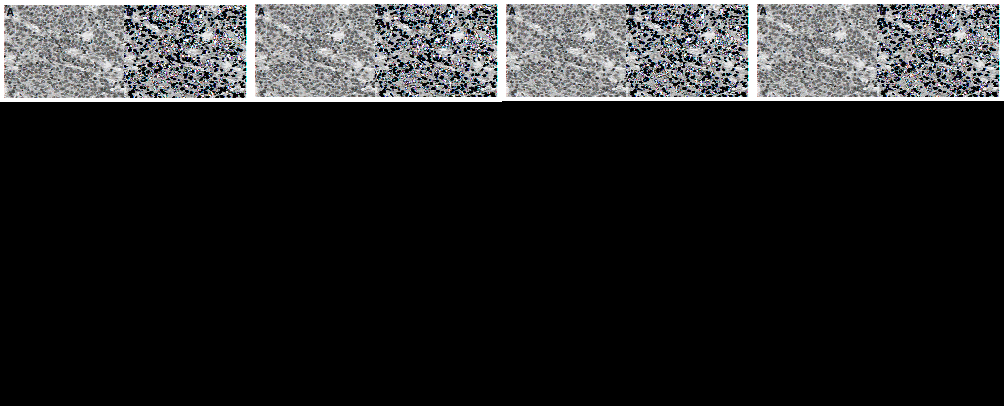

Supplement: Supplementary file 1 — Supplementary figure 1 [file 41416_2019_429_MOESM1_ESM.tif]

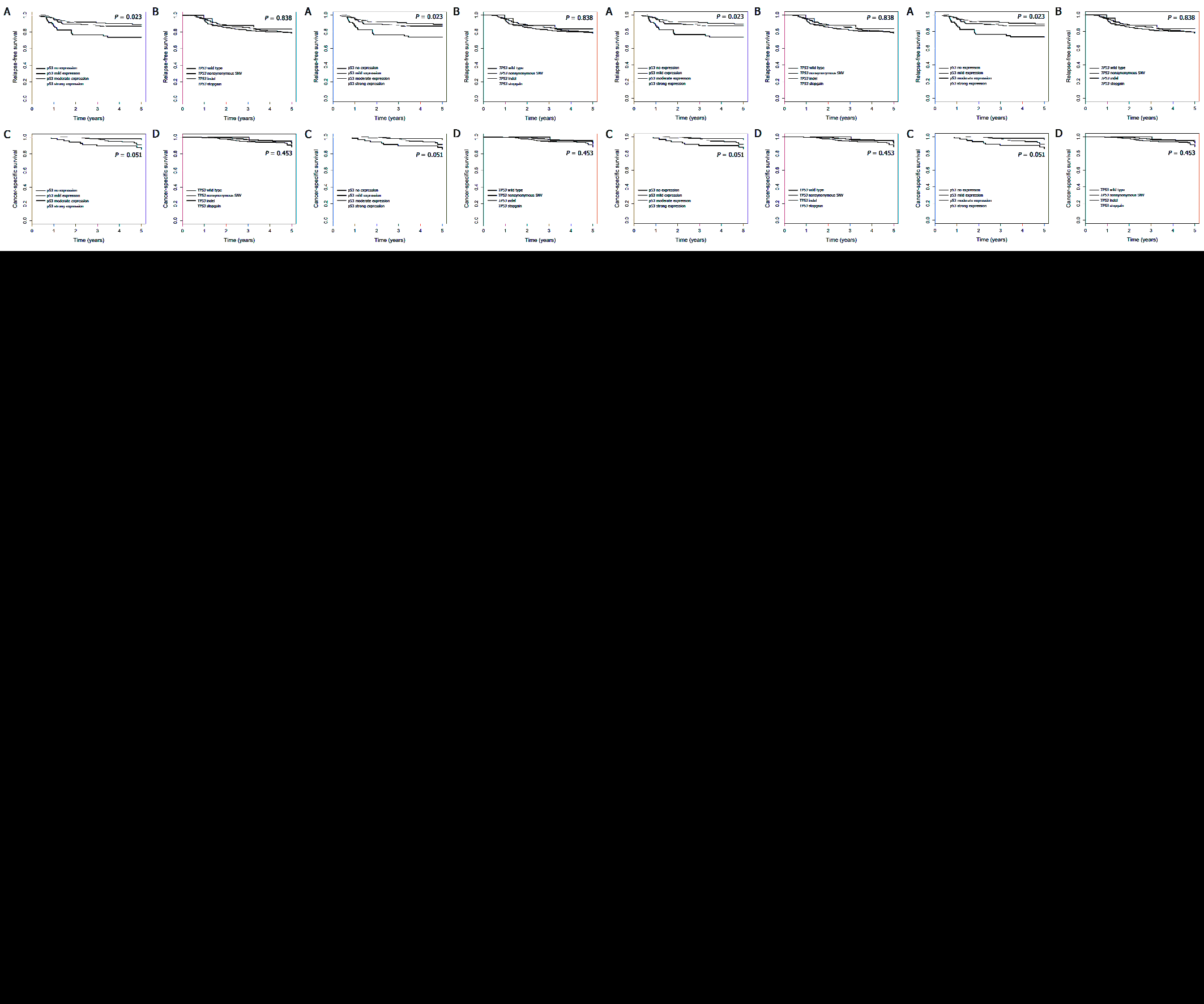

Supplement: Supplementary file 2 — Supplementary figure 2 [file 41416_2019_429_MOESM2_ESM.tif]

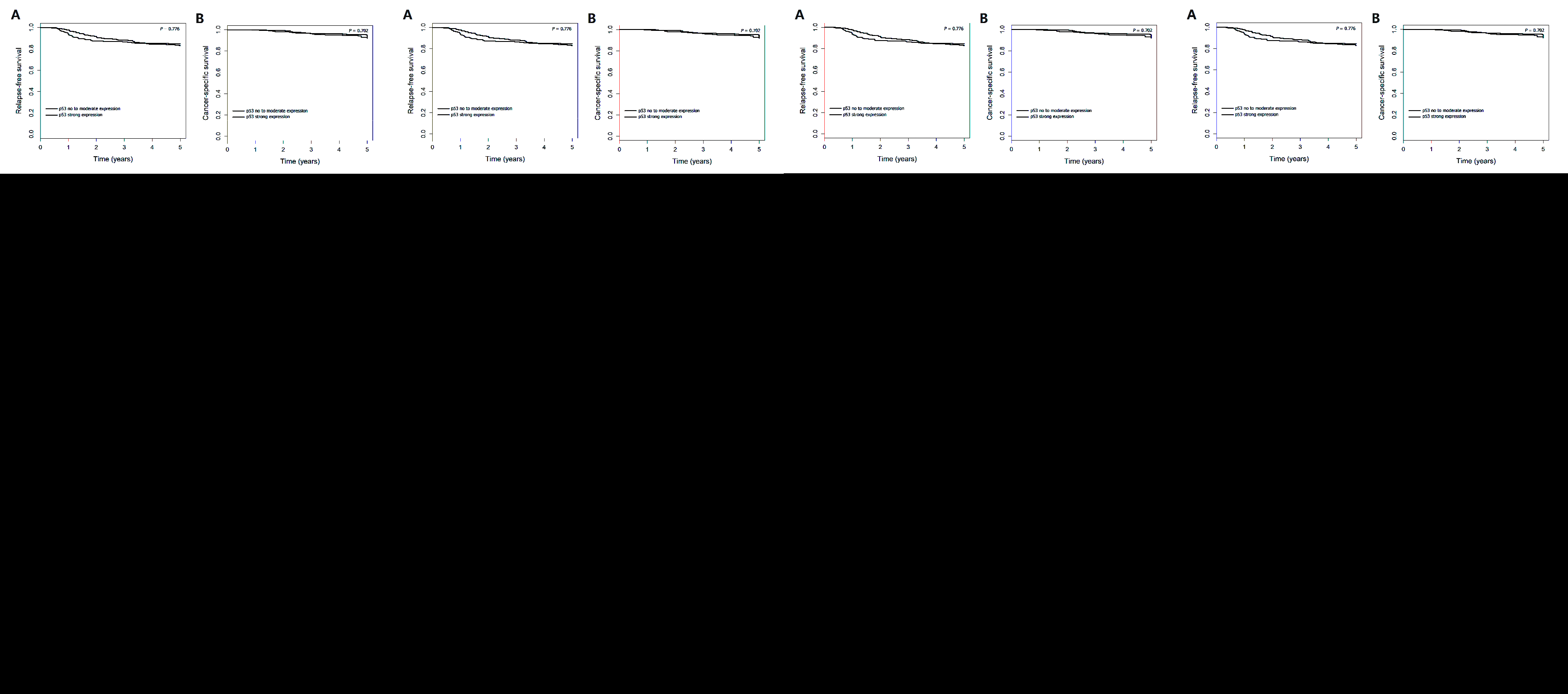

Supplement: Supplementary file 3 — Supplementary figure 3 [file 41416_2019_429_MOESM3_ESM.tif]

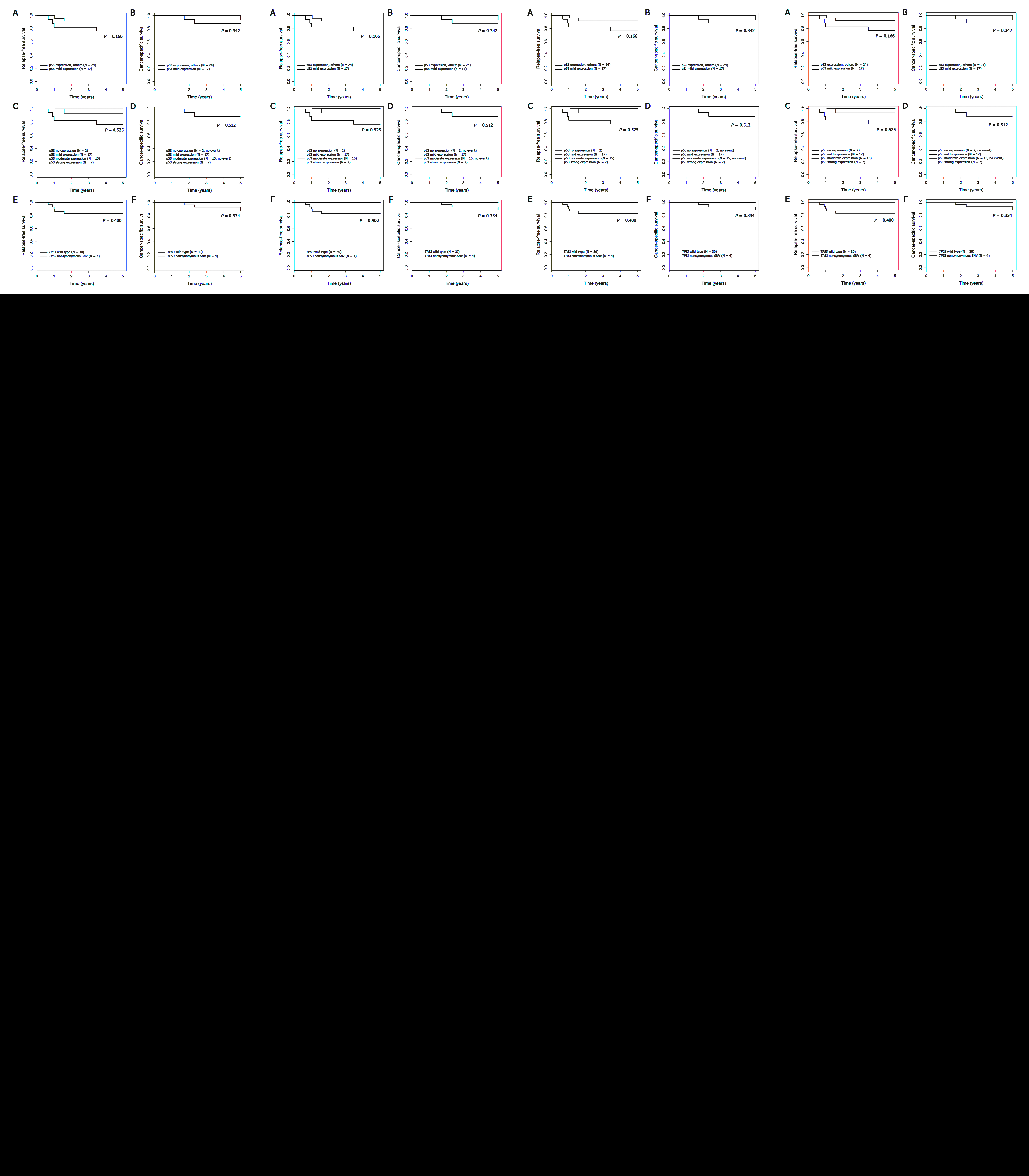

Supplement: Supplementary file 4 — Supplementary figure 4 [file 41416_2019_429_MOESM4_ESM.tif]

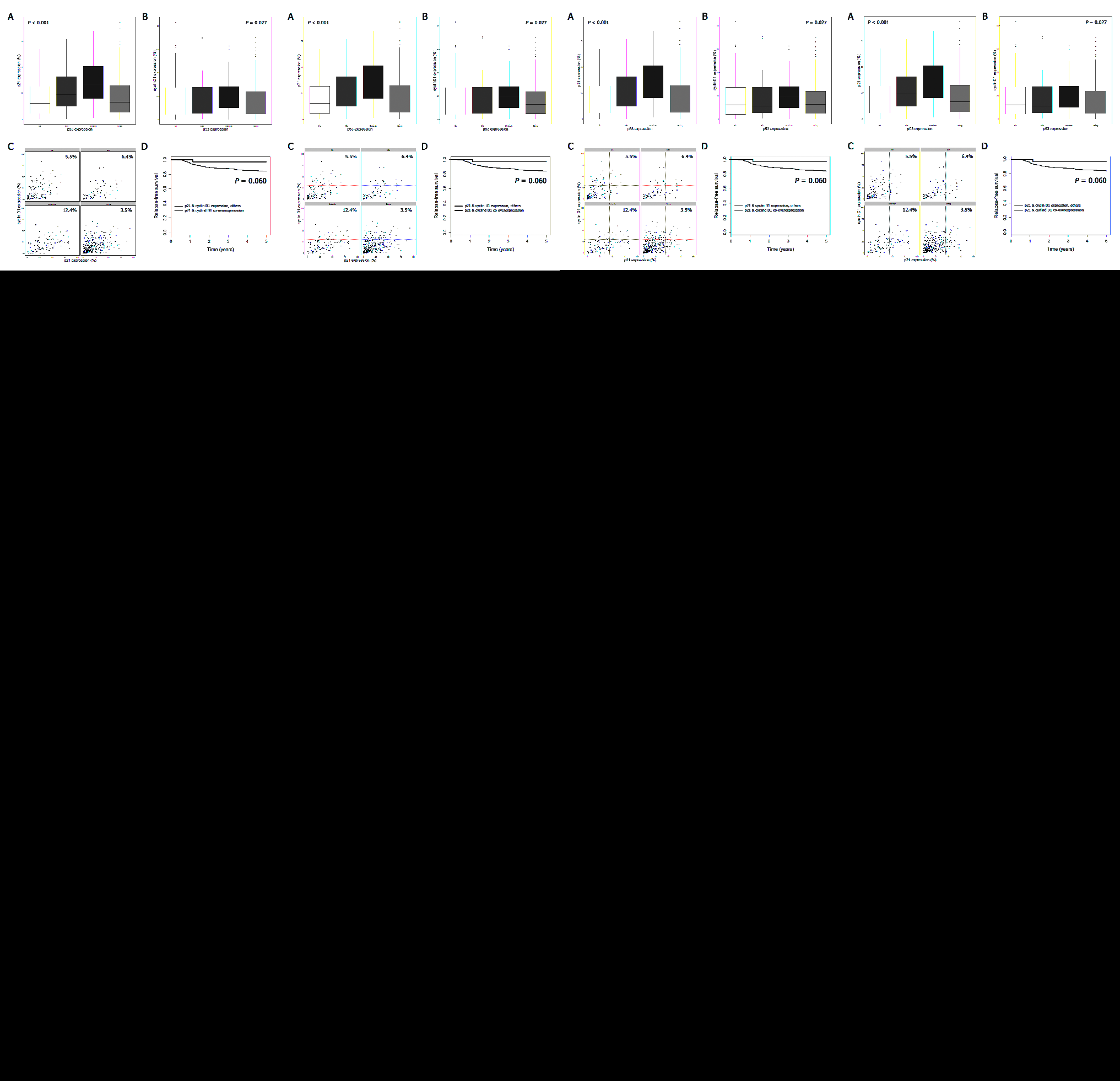

Supplement: Supplementary file 5 — Supplementary figure 5 [file 41416_2019_429_MOESM5_ESM.tif]
