# Supplementary material for: p53 expression status is associated with cancer-specific survival in stage III and high-risk stage II colorectal cancer patients treated with oxaliplatin-based adjuvant chemotherapy
Source: Br J Cancer. 2019 Mar 21;120(8):797–805. doi: 10.1038/s41416-019-0429-2 (PMC6474280; doi:10.1038/s41416-019-0429-2)
Supplement: Supplementary file 7 — Article File [file 41416_2019_429_MOESM7_ESM.docx]

**Supplementary figure legends**

Supplementary figure 1. Quantitative analysis of p53 immunohistochemistry. (A) Raw image of p53 immunohistochemistry, and (B) Annotation of the staining intensity of p53 (blue: 0, yellow: 1+, orange: 2+, and red: 3+ according to the Nuclear v9 algorithm).

Supplementary figure 2. Detailed Kaplan-Meier survival curves. (A) 5-year relapse-free survival (RFS) according to the four p53 expression subgroups, (B) 5-year RFS according to TP53 genotype, (C) 5-year cancer-specific survival (CSS) according to the four p53 expression subgroups, and (D) 5-year CSS according to TP53 genotype.

Supplementary figure 3. Kaplan-Meier survival curves and the conventional classification of p53 expression. (A) 5-year relapse–free survival according to the p53 expression subgroups (no to moderate (<50%) and strong (≥50%), (B) 5-year cancer-specific survival according to the p53 expression subgroups (no to moderate (<50%) and strong (≥50%).

Supplementary figure 4. Survival analysis in 41 microsatellite instability-high colorectal cancers. (A) 5-year relapse-free survival (RFS) according to p53 expression, (B) 5-year cancer-specific survival (CSS) according to p53 expression, (C) 5-year RFS according to the four p53 expression subgroups, (D) 5-year CSS according to the four p53 expression subgroups, (E) 5-year RFS according to *TP53* genotype, and (F) 5-year CSS according to *TP53* genotype.

Supplementary figure 5. Nuclear p21 and cyclin D1 expression in four p53 expression subgroups of colorectal cancers. (A) Box-plot of nuclear p21 expression, (B) Box-plot of nuclear cyclin D1 expression, (C) Scatter plot according to p21 and cyclin D1 expression, and (D) 5-year relapse-free survival according to p21 and cyclin D1 co-expression status.
